# Association of Female Sexual Dysfunction and Fertility: a cross sectional study

**DOI:** 10.1186/s40738-019-0065-9

**Published:** 2019-11-23

**Authors:** Felix Mwembi Oindi, Alfred Murage, Valentino Manase Lema, Abraham Mwaniki Mukaindo

**Affiliations:** Department of Obstetrics and Gynaecology, Aga Khan University Hospital, Nairobi, Kenya

**Keywords:** Female sexual dysfunction, Subfertility, Female sexual function index, Questionnaire.

## Abstract

**Background:**

Sexual function plays an essential role in the bio-psychosocial wellbeing and quality of life of women and disturbances in sexual functioning often result in significant distress. Female sexual dysfunction (FSD) and subfertility are common problems affecting approximately 43 and 20% of women respectively. However, despite the high prevalence of both conditions, little has been studied on the effects of subfertility on sexual functioning especially in sub-Saharan Africa. We set out to compare the prevalence of female sexual dysfunction in patients on assessment for sub-fertility and those either seeking or already on fertility control services at a private tertiary teaching hospital in Kenya.

**Methods:**

This was an analytical cross sectional study. Eligible women of reproductive age (18–49 years), attending the gynaecological clinics with complaints of subfertility and those seeking fertility control services were requested to fill a general demographic tool containing personal data and the Female Sexual Function Index (FSFI) questionnaire after informed consent. Prevalence of sexual dysfunction was calculated as a percentage of patients not achieving an overall FSFI score of 26.55. Univariate and multivariate analysis were done to compare clinical variables to delineate the potential association.

**Results:**

The prevalence of female sexual dysfunction was 31.2% in the subfertile group and 22.6% in fertility control group. The difference was not statistically significant (*p* = 0.187). The mean domain and overall female sexual function scores were lower in the subfertile group than the fertility control group though this was not statistically significant. The most prevalent sexual domain dysfunctions in both the subfertility and fertility control groups were desire and arousal while the least in both groups was satisfaction dysfunction. Subfertility type was not associated with sexual dysfunction. Higher education attainment was protective of female sexual dysfunction in the subfertile group while use of hormonal contraception was associated with greater sexual impairment in the fertility control group. On logistic regression analysis, higher maternal age and alcohol use appeared to be protective against sexual dysfunction.

**Conclusion:**

The present study demonstrated no association between the fertility status and the prevalence female sexual dysfunction. Subfertility type was not associated with sexual dysfunction. Education level and hormonal contraception use were associated with female sexual dysfunction in the subfertile and fertility control groups respectively while alcohol use and higher maternal age appeared to be protective against sexual dysfunction.

## Plain language summary

Female sexual dysfunction is a common condition affecting approximately 43% of women. Subfertility is also common affecting approximately 1 in 5 couples. This study aimed to compare sexual function between patients on fertility assessment and those seeking family planning services. A questionnaire (Female sexual function index questionnaire) was used to the collect the patients’ data and sexual history which was then analyzed.

Of the 186 respondents (93 in the subfertility group and 93 in the family planning group), 50 had impaired sexual function. Of these, 29 were from the subfertility group while 21 were from the family planning group. The most affected sexual function domains in both groups were desire and arousal while the least affected was satisfaction.

In the fertility control group, women with higher education attainment had less sexual dysfunction while in the family planning group, women on hormonal contraception had more sexual dysfunction. Women who used alcohol and those above 35 years of age had a lesser sexual dysfunction.

In conclusion, being subfertile was not associated with female sexual dysfunction. Maternal age, education level, use of alcohol and use of hormonal contraception affected sexual function and need to be explored further.

## Background

Sexual function plays a central role in the biopsychosocial wellbeing and quality of life of human beings [1]. It encompasses issues such as sex, reproduction, pleasure, intimacy, erotism, identity and gender roles, and sexual orientation [2] and is under the dynamic interaction of the physical, economic, religious, psychological and emotional factors [2, 3]. Any impairment in sexual function could have detrimental effects on the quality of life of the affected subjects [4].

Female Sexual Dysfunction (FSD) is defined as a “disorder of sexual desire, arousal, orgasm, and sexual pain that results in significant personal distress. It is a multifactorial, age-related and progressive problem” [5, 6] that is based on disturbances to the female sexual response.

Female sexual dysfunction affects approximately 43% of women according to data from the National Health and Social Survey (1992; *n* = 1749; 18–59 years old women) [7]. It is more prevalent in women with chronic ailments, genital atrophy, history of sexual abuse and those with psychosocial stressors such as subfertility and poor interpersonal relationships [8]. In particular, women with subfertility have been shown to have up to one and half times greater prevalence of sexual dysfunction than their fertile counterparts [9, 10].

Subfertility is an “emotional rollercoaster” to affected couples more so women with resultant mental stress [11]. It is a significant life stressor and might negatively impact on sexual function. The psychological stress of subfertility which would stem from the societal perception of the sub-fertile ‘woman’ who may be considered to be undergoing a punishment from a previous behavior like premarital sex or abortion, or from the physical pressure to conceive may result in new or aggravation of existing sexual dysfunction. The subfertile couple is more prone to depression, anxiety and stress [12, 13]. The increased stress levels would adversely affect the marital satisfaction and adversely affect their sexual health. On the other hand, the medical procedures for investigation or treatment of subfertility may arouse a sense of anxiety and hence affect sexual functioning [12].

Whether sexual dysfunction is the cause or consequence of subfertility is difficult to establish. For instance, sexual dysfunction might result in decreased coital frequency compounding the issue of subfertility due to reduced exposure. On the other hand, the psychological pressure to get pregnant stemming from sex on demand could result in a reduction in enjoyment of sex aggravating sexual dysfunction. Indeed, situational sexual dysfunction and loss of a couple’s intimacy may occur as a consequence of timed intercourse where focus for coitus is no longer pleasure but conception [10]. Therefore, the relationship between subfertility and sexual function might be bidirectional and need to be addressed for adequate management of either problem. Early diagnosis and treatment of sexual dysfunction among this group of patients might improve outcomes of subfertility treatment.

Data on sexual dysfunction in sub-Saharan Africa, especially Kenya is scarce. This was, to the best of our knowledge, the first study evaluating the prevalence of female sexual dysfunction among patients on follow up for subfertility in the region. As a result, we set out to compare sexual function among patients on subfertility assessment and those seeking fertility control services at a private tertiary teaching hospital in Kenya. The choice for fertility control clients was that they are presumably sexually active and having normal sexual functioning, hence seeking contraception to avoid a pregnancy. The study of female sexual dysfunction is particularly important in our society where the topic of sexuality is considered a taboo by many and many patients may not freely discuss such issues.

## Methodology

### Objective

To compare the prevalence of sexual dysfunction, as measured by the FSFI-Q, between patients on assessment for subfertility and those either seeking or on various contraceptive methods at the Aga Khan University Hospital, Nairobi.

### Study design

This was an analytic cross sectional study.

### Study setting and participants

The study was conducted at the Aga Khan University Hospital Gynaecology outpatient Clinics which run on every weekday. Both new and old patients on various stages of fertility assessment or treatment and those presenting for or already on a contraceptive method were approached and assessed for eligibility. Eligible participants who provided informed consent were recruited. A sub-fertile patient was defined as one with inability to conceive after at least 12 months of regular unprotected coitus [12]. The eligibility criteria was as follows:

#### Inclusion criteria was

Women 18–49 years of age attending the gynaecology outpatient clinic with subfertility and those either seeking or already on a family planning method who were sexually active in the preceding 4 weeks and had English literacy as the questionnaire was self-administered in English and due to unavailability of a validated form of the FSFI-Q in the Kiswahili language commonly spoken in Kenya.

#### Exclusion criteria was

Pregnant patients and those in the puerperium; those with gynaecologic conditions like malignancies, fistula, chronic pelvic pain, genital prolapse and lower genital tract abnormality; those with medical conditions associated with sexual dysfunction like diabetes, hypertension, endocrine disorders and psychiatric illnesses and those who previously had pelvic floor surgery were excluded due potential effect of these conditions on sexual function.

### Study procedures and tools

Patients attending the gynaecology clinic with subfertility and those presenting for or already on contraception were approached by the principal investigator or the research assistants for eligibility. Eligible participants were then requested to fill the two data collection tools, the FSFI-Q and the general demographic tool, after an explanation and giving informed consent. The demographics tool collected information on the age, parity (and previous pregnancy outcomes), BMI and other associated factors that might impact on sexual function. Some of these included the partner’s age, educational level, marital status, contraceptive use (and type), alcohol and substance use and abuse, history of sexual abuse, and social support. These tools were self-administered and the participants were recruited by convenience sampling. Sexual function was measured using the domains in the FSFI-Q with those with overall scores below 26.55 being considered to have impaired sexual functioning [2]. The participants who wished to know their FSFI scores were informed by phone and those with scores below 26.55 were advised to attend the sexual health clinic for further assessment and management. None of the approached participants declined taking part in the study.

### Sample size calculation and sampling method

The baseline prevalence level for sexual dysfunction in the general population was assumed to be 43% based on a prior study [7]. It is estimated that subfertile patients experience up to 50% greater sexual dysfunction (i.e. 64.5%) than their general counterparts [9, 10]. Sexual function in women on various fertility control services is not statistically different from that of the general population [14–16] hence women seeking fertility control were used as a control group. There being no local data on female sexual dysfunction in the general population let alone in subfertile patients, we assumed the prevalence as reported in literature and utilized it in the calculation of the sample size. Sample size calculation was done using the formula for comparing two proportions:

*n* = (Z_α/2_ + Z_β_)^2^ * (p_1_(1-p_1_) + p_2_(1-p_2_)) / (p_1_-p_2_)^2^.

Where:

*n* = required sample size per group.

Z_α/2_ = the critical value of the Normal distribution at α/2 (For a confidence level of 95%, α is 0.05 and the critical value is 1.96).

Z_β_ = the critical value of the Normal distribution at β (For a power of 80%, β is 0.2 and the critical value is 0.84).

P_1_ and P_2_ = the expected sample proportions of the two groups.

P_1_ = 0.43 P_2_ = 0.43 + 0.5(0.43) =0.645.

Therefore:

*n* = (1.96 + 0.84)^2^*(0.645(1–0.645) + 0.43(1–0.43)/ (0.645–0.43)^2^.

*n* = 93.

Total sample size = n*2 = 93*2 = 186.

### Data management and analysis

The prevalence of sexual dysfunction was determined as the percentage of patients with domain and overall scores below the cut-off levels. Chi-square test was used to test the association between the categorical variables. The patients’ socio-demographics characteristics were compared to determine any association between the patients with sexual dysfunction and those without sexual dysfunction. Univariate analysis was done to evaluate the relationship of each socio-demographic characteristic with sexual dysfunction in the sub-fertile group. The same was done for the subjects seeking or on fertility control services in order to delineate the potential pattern of association. Univariate logistic regression analysis were conducted for variables with potential confounding effect on sexual dysfunction. Logistic regression models were constructed after which logistic regression was performed for an adjusted odds ratio for each of the factors. Data values were expressed as mean ± SD, count (%) and odds ratio. *P* < 0.05 was considered statistically significant. Data analysis was performed using STATA version 12.0 and the data expressed in tables and graphs.

### Ethical considerations

Ethical approval was sought from the Aga Khan University Ethics Committee before commencing the study. Prior to being involved in the study, the participants gave a written informed consent. Patients were informed that they had a right to refuse or withdraw from the study at any point and this would not impact on the quality of care received subsequently. Patient confidentiality and privacy was maintained during the entire study period with use of number identifiers alongside safe and restricted data storage. The data collection forms were safely kept in a locked cabinet to which only the primary investigator and the research assistants had access to. Subjects with sexual dysfunction were referred to the sexual health clinic for further evaluation and management. None of the study participants aged below 18 years.

### The female sexual function index questionnaire (FSFI-Q)

The FSFI-Q is a multidimensional self-report tool for assessing key dimensions of female sexual functioning over the preceding 4 weeks [17, 18]. This standardized questionnaire described by Rosen and colleagues [17] consists of 19-items that assess six domains of female sexual functioning. The domains include: sexual desire (items 1 and 2), arousal (items 3–6), lubrication (items 7–10), orgasm (items 11–13), satisfaction (items 14–16) and sexual pain (items 17–19). Each of the items has a Likert scale score ranging from 0 to 5 and each of the 6 domains’ scores are calculated by adding the scores of the individual items that comprise the domain and multiplying by a respective domain factor which homogenizes each dimension’s influence. The full scale or total FSFI score ranges from 2 to 36 and is the sum of all the scores in the six domains [2, 4]. Higher scores indicate a better sexual functioning with a 26.55 or less cut off score indicative of sexual dysfunction according to a validation study [2]. Equally, each of the domains has a cut-off for sexual dysfunction.

## Results

### Patient characteristics

A total of 186 women were recruited by convenience sampling over the study duration (November 2015 to November 2016). Of these, 93 had presented with subfertility and were at various stages of fertility assessment while the other 93 had presented either for fertility control services or were already on a fertility control service. The mean age of the participants was 32.4 (SD 5.79) with the biggest group (58.6%) being the 30–39 age group. Similarly, a great proportion of the partners were below 40 years (68.9%). Majority of the women were married (81.7%) and had a university education (90.3%). The study participants were mostly non-obese (80.7%) and majority reported not using alcohol (64.5%). Only 4 patients (2.2%), 2 in the subfertility and 2 in the fertility control groups, reported a history of smoking while only 2 (1.1%) reported a history of rape (both being in the subfertility group). However, 6 patients (3.2%) had missing data on the history of rape. Three of these were from the subfertility group while 3 were from the fertility control group.

The subfertile and fertility control subjects did not differ significantly in terms of body mass index (BMI), previous miscarriage, education level, frequency of coitus and history of alcohol use. We would not adequately compare those with a smoking history or rape. The two groups however exhibited significant differences in their ages, partner’s age, marital status and previous live birth (Table 1). The subfertile subjects and their partners were more likely to be older and married but less likely to have had a previous live birth when compared to the fertility control subjects.

**Table 1:** Socio-demographic characteristics of the subfertile and fertility control subjects

Parameter	Subfertile group (*n* = 93)	Fertility control group (n = 93)	*P*-value
Age (years)			
20–29 (%)	18 (19.35)	37 (39.78)	
30–39 (%)	56 (60.22)	53 (56.99)	
40–49 (%)	19 (20.43)	3 (3.23)	
Mean ± SD	34.20 ± 5.72	30.51 ± 5.27	**< 0.001**^**a**^
Body Mass Index (Kg/m^2^)			
Underweight (< 18)	1 (1.08)	2 (2.15)	
Normal weight (18–24.9)	32 (34.41)	35 (37.63)	
Overweight [19–24]	42 (45.16)	38 (40.86)	
Obese (> 30)	18 (19.35)	18 (19.35)	
Mean ± SD	26.72 ± 4.24	26.14 ± 4.35	0.358^a^
Partner Age (years)			
< 40	59 (63.44)	71 (76.34)	
≥ 40	34 (36.56)	22 (23.66)	
Mean ± SD	37.73 ± 8.28	34.14 ± 5.72	**< 0.001**^**a**^
Marital Status (married vs unmarried)			**< 0.001**^**b**^
Married	89 (95.70)	63 (67.74)	
Married ≤3 years	25 (26.88)	23 (36.51)	0.385^b^
Married > 3 years	65 (69.89)	40 (63.49)	
Mean ± SD	6.79 ± 4.66	6.10 ± 4.32	
Single	4 (4.3)	30 (32.3)	
Previous live birth			**< 0.001**^**b**^
Present	33 (35.48)	60 (64.52)	
None	60 (64.52)	33 (35.48)	
Previous miscarriage			0.130^b^
Yes	35 (37.63)	25 (26.88)	
None	58 (62.37)	68 (73.12)	
Education Level			0.621^b^
< 8 years (Primary)	3 (3.23)	1 (1.08)	
8–12 years (Secondary)	6 (6.45)	7 (7.53)	
> 12 years (College)	84 (90.33)	85 (91.4)	
Frequency of coitus			0.760^b^
≤ 10 per month	43 (46.24)	46 (49.46)	
> 10 per month	50 (53.76)	47 (50.54)	
History of alcohol use			0.123^b^
Yes	28 (30.11)	38 (40.86)	
None	65 (69.89)	55 (59.14)	

^a^Student’s t-test; ^b^Chi square test

### Female sexual dysfunction prevalence as per female sexual function index questionnaire (FSFI-Q)

Using a cutoff score of 26.55 on the FSFI-Q, the prevalence of female sexual dysfunction (FSD) was 26.9% among the combined study subjects. On the other hand, the prevalence of FSD was 31.2% (*n* = 29 of 93) and 22.6% (*n* = 21 of 93) in the subfertile and fertility control groups respectively which was not statistically significant (*p* = 0.187). Moreover, despite the subfertile group portraying lower mean overall and domain scores, these were not statistically significant from the fertility control group (Table 2).

**Table 2:** Mean domain-specific scores and overall sexual index (fsfi) questionnaire scores in women presenting with subfertility and for fertility control

FSFI Domain	Subfertility (*n* = 93)	Fertility control (*n* = 93)	Maximum score	*P*-value
Desire	3.85 ± 0.97	3.97 ± 0.92	6.0	0.388^a^
Arousal	4.37 ± 1.03	4.62 ± 0.85	6.0	0.073^a^
Lubrication	5.03 ± 0.99	5.17 ± 0.99	6.0	0.336^a^
Orgasm	4.69 ± 1.27	4.86 ± 1.11	6.0	0.332^a^
Satisfaction	5.02 ± 1.11	5.32 ± 0.87	6.0	**0.042**^**a**^
Pain	4.93 ± 1.26	5.12 ± 1.01	6.0	0.258^a^
Total Sexual function Score	27.86 ± 5.14	29.14 ± 3.82	36.0	0.056^a^
Number (%) of women with FSFI score of < 26.55	29 (31.2%)	21 (22.6%)		0.187^b^

Data expressed as mean ± SD

^a^Student’s t-test ^b^Chi square test

**Fig. 1:**
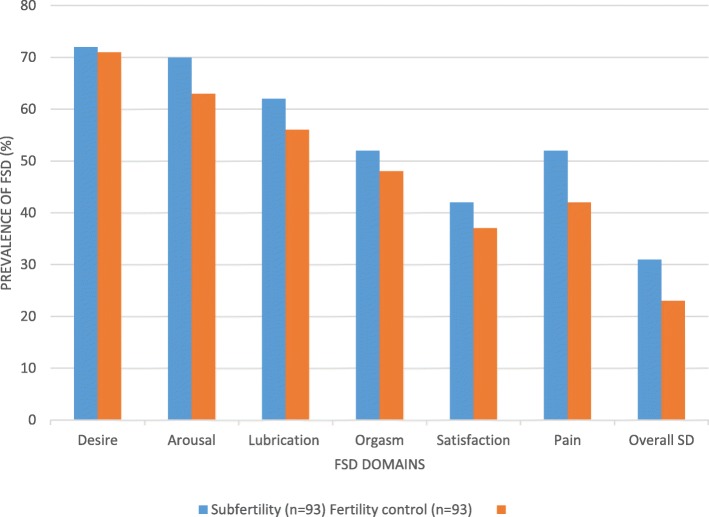
Prevalence of Female Sexual Dysfunction in the study population

The most affected domains in both the subfertility and fertility control groups were desire and arousal while the least affected in both groups was the satisfaction domain. The proportion of those with sexual dysfunction in all the domains and total FSF score was higher in the subfertility group than the fertility control group though none was statistically significant (Fig. 1).

There were 40 women with primary subfertility and 53 with secondary subfertility. The prevalence of FSD in the primary and secondary subfertile women was 32.5% (*n* = 13) and 30.2% (*n* = 16) respectively which was not significantly different (*p* = 0.057).

### Analysis of factors associated with female sexual dysfunction

On evaluation of the association between the various socio-demographic variables and sexual dysfunction among the subfertility group, only education level was significantly associated with sexual dysfunction (Table 3). Women with college level education were less likely to have sexual dysfunction when compared to those with no college education. The duration of subfertility (≤3 years vs > 3 years) did not appear to affect sexual function (OR 0.82, CI 0.34–1.96, *p* = 0.666).

**Table 3:** Association of variables to female sexual dysfunction (fsd) in the subfertility group (*n* = 93)

Variable	Categories	FSD Prevalence (%)	OR (95% CI)	*P*-value^b^
Age (Years)	< 35	36.4	1	0.197
	≥35	23.7	0.54 (0.21–1.39)	
Partner age (Years)	< 40	35.6	1	0.229
	≥40	23.5	0.56 (0.21–1.47)	
BMI (Kg/m^2^)	Not obese	30.7	1	0.827
	Obese	33.3	1.13 (0.38–3.40)	
Marital status	Not married	50	1	0.408
	Married	30.3	0.44 (0.06–3.32)	
Duration of marriage (years)	≤3	28	1	0.799
	> 3	30.8	1.14 (0.41–3.19)	
Previous live birth	None	33.3	1	0.548
	Yes	27.3	0.75 (0.29–1.92)	
Previous miscarriage	None	34.5	1	0.379
	Yes	25.7	0.66 (0.26–1.68)	
Education level	No college	70	1	**0.005**
	College	26.5	0.15 (0.34–0.70)	
Frequency of coitus/ month	≤10	32.6	1	0.791
	> 10	30	0.88 (0.37–2.15)	
Alcohol use	None	37.1	1	0.140
	Yes	21.4	0.46 (0.16–1.33)	

^b^Chi square test

For the fertility control group, only the use of hormonal contraception was significantly associated with sexual dysfunction (Table 4). The duration of marriage (≤3 years vs > 3 years) among the married (*n* = 63 (67.7%)) did not appear to be significantly associated with female sexual dysfunction (OR 1.42, CI 0.37–5.29, *p* = 0.602)

**Table 4:** Association of variables to female sexual dysfunction (fsd) in the fertility control group (*n* = 93)

Variable	Categories	FSD Prevalence (%)	OR (95% CI)	*P*-value^b^
Age (Years)	< 35	24.3	1	0.495
	≥35	17.4	0.66 (0.19–2.22)	
Partner age (Years)	< 40	25.4	1	0.334
	≥40	15	0.52 (0.13–2.01)	
BMI (Kg/m^2^)	Not obese	22.7	1	0.967
	Obese	22.2	0.97 (0.28–3.38)	
Marital status	Not Married	23.2	1	0.905
	Married	22.2	0.94 (0.33–2.65)	
Previous live birth	None	21.7	1	0.777
	Yes	24.2	0.86 (0.31–2.38)	
Previous miscarriage	None	25.4	1	0.343
	Yes	16	0.56 (0.17–1.89)	
Education level	No college	25	1	0.865
	College	22.4	0.86 (0.16–4.68)	
Frequency of coitus/ month	≤10	21.4	1	0.825
	> 10	23.4	1.12 (0.41–3.06)	
Alcohol use	None	26.9	1	0.116
	Yes	13.2	0.41 (0.13–1.29)	
Contraceptive use	Non hormonal	33.3	1	**0.006**
	Hormonal	29.2	4 (1.38–11.62)	

^b^Chi square test

Stepwise regression modelling was employed to evaluate the association of the socio-demographic variables with FSD. The initial step assessed each of the variable’s association with subfertility. Variables found to be significantly associated with subfertility were age (OR 2.10, CI 1.11–3.98, *p* = 0.020), previous live birth (OR 0.30, CI 0.16–0.57, p = < 0.001), marital status (OR 10.6, CI 3.27–34.29, p = < 0.001) and partner’s age (OR 2.04, CI 1.06–3.96, *p* = 0.030)

The variables were then assessed for their association with sexual dysfunction. The variables found to be significantly associated to sexual dysfunction were education level (OR 0.32, CI 0.12–0.88, *p* = 0.02) and alcohol use (OR 0.42, CI 0.19–0.90, *p* = 0.021). From the two steps, no variable emerged to be a potential confounder given that none was significantly associated with both subfertility and sexual dysfunction. Subfertility was not associated with sexual dysfunction (Crude OR 1.55, CI 0.80–3.00, *p* = 0.187)

From the logistic regression, only alcohol use and higher maternal age affected sexual function with both appearing to be protective (Table 5)

**Table 5:** Logistic regression analysis of the odds ratio of sexual dysfunction of each risk factor adjusted for other variables in the model

Risk factor	Adjusted OR (95%)	*P*-value
Subfertility	1.87 (0.92–3.80)	0.085
Age	0.41 (0.18–0.91)	**0.028**
Alcohol use	0.40 (0.18–0.89)	**0.024**
Education level	0.35 (0.12–1.03)	0.058

## Discussion

Sexual dysfunction is a common problem which can negatively affect a woman’s self-esteem, quality of life and interpersonal relationships. However, its burden has not been adequately assessed especially in sub-Saharan Africa. This study demonstrated that 26.9% of the study participants had sexual dysfunction. These findings are comparable to other studies showing a sexual dysfunction prevalence of 26–28% among reproductive age women [18, 25].

The prevalence reported in the present study is lower than that reported in other population based studies [4, 7, 19–21]. These studies however included menopausal women and had a lower number of women attaining above college level education. Our study population included only reproductive age women and had higher education attainment (90.3% had college level education). Advancing age is associated with greater sexual dysfunction especially after menopause [21]. On the other hand, higher education has been shown in other studies to be protective of sexual dysfunction [2, 19]. For instance, Safarinejad (2006) showed a prevalence rate of sexual dysfunction among Iranian women of 31.5%. The study participants however included menopausal women (range 20–60 years) with only 38.8% having above high school education. In Egypt, Ibrahim et al. (2013) found a 52.8% prevalence of sexual dysfunction. However, majority (51.3%) were post-menopausal and 71% had undergone female genital mutilation (FGM) hence the higher prevalence as advanced age adversely affects sexual function and possibly female FGM especially if type II or III. Moreover, only 15.2% had college education.

The primary aim of our study was to compare the prevalence of sexual dysfunction between patients on follow up for subfertility and those seeking fertility control services. We found a prevalence of sexual dysfunction of 31.2 and 22.6% in the subfertility and fertility control groups respectively. Despite the apparent difference in the prevalence of sexual dysfunction in the two groups, analysis of data from the study demonstrated no statistically significant difference in the prevalence of sexual dysfunction between subfertility and fertility control subjects (adjusted odds ratio 1.87, CI 0.92–3.80, *p* = 0.085). The subfertile group had lower mean total FSFI and domain scores though only the satisfaction score was statistically significant from the fertility control group. The decreased satisfaction is possibly due to low self-esteem and poor body-image as a result of or as a cause of the subfertility. Moreover, the psycho-social pressures to conceive stemming from “sex-on-demand” might result in loss of couple intimacy [9]. The absence of significant difference in sexual function could be due to fact that the study was conducted in a tertiary private hospital whose clientele are more likely to be of a higher socioeconomic status with higher education attainment making them more aware of their own bodies and hence sexual performance.

Studies on the association between subfertility and female sexual dysfunction have reported conflicting results. Iris et al. (2013) and Furukawa et al. (2012) found no significant difference in the prevalence of sexual dysfunction between subfertile and fertile women [22, 23], similar to the present study findings. Iris et al. (2013) in their study (*n* = 809) with 174 being subfertile, demonstrated a significantly greater prevalence of sexual dysfunction with increasing duration of subfertility though there was no significant difference in sexual dysfunction between the subfertile and fertile groups [22]. However, this study by was not powered to detect the difference in sexual dysfunction between subfertile and fertile groups and excluded women with secondary subfertility, a known risk factor for sexual dysfunction [13]. The present study found no significant association of sexual dysfunction with subfertility duration. On the other hand, Furukawa et al. (2012) in a study comparing the rate of dyspareunia and sexual dysfunction among women seeking fertility services found no significant association between subfertility and sexual function (adjusted odds ratio 1.44, CI 0.77–2.69, *p* = 0.25). Moreover, just like the present study findings, the subfertile subjects were more likely to be married [23].

The findings of lower total and individual domain FSFI scores among subfertility patients have also been reported in other studies [9, 24, 26]. To determine the effect of subfertility on sexual function, Ashraf et al. studied 384 Iranian women divided in two groups (fertile and subfertile). Using the FSFI, the mean sexual function scores were significantly lower in the subfertile group [24]. However, only 47.4% of their subfertility subjects had college level education unlike our subjects (90.3%). Similarly, Tanha et al., (2014) demonstrated a significantly lower individual domain and total FSFI scores in the subfertile subjects in comparison with the controls [24] while Mirblouk et al., (2016) found a significantly greater occurrence of sexual dysfunction among the subfertile subjects [26]. Milheiser et al., compared 119 subfertile and 99 fertile women and found a 40% prevalence of sexual dysfunction in the subfertile group compared to 25% in the fertile group with the subfertile group having significantly lower mean total, desire and arousal scores. Interestingly, they found a significantly lower frequency of coitus among the subfertile group [9]. This is unusual for subfertile subjects who are expected to have more frequent intercourse in order to increase their fecundability. In the present study, the frequency of coitus was not significantly associated with sexual dysfunction.

In the current study, the most common alterations in both the subfertile and fertility control groups were lack of desire and arousal. These finding are similar to those by Mirblouk et al. (2016) that demonstrated a greater occurrence of desire, arousal and orgasmic dysfunctions [27]. A high correlation between sexual desire and arousal make the desire and arousal disorders to be among the commonest complaints in clinical practice. Aggarwal et al. (2013) similarly found arousal dysfunction to be the most prevalent among the subfertile women (70%) while desire and orgasmic dysfunctions were the most prevalent in the fertility group each at 40% [10]. Moreover, just like the present study, the prevalence arousal, lubrication and pain dysfunctions was higher in the subfertility group. The findings differed with the present study by demonstrating a lower prevalence of desire, orgasmic and satisfaction dysfunctions in the subfertility group. Khademi et al. (2008) demonstrated an 80% prevalence of arousal dysfunction and a 22% orgasmic dysfunction among subfertility women [28].

Secondarily, the present study sought to assess the difference in sexual function between subjects with primary and those with secondary subfertility. There was no statistically significant difference in sexual dysfunction between subjects with primary and secondary subfertility. These findings are similar to those by Kabil et al. (2015) and Keskin et al. (2011). Kabil and colleagues examined the effects of subfertility aetiology and depression on the female sexual function. They compared sexual function among 83 and 59 women with primary and secondary subfertility respectively and did not find any difference in FSD between the two groups [12]. Similarly, an Iranian study by Tanha et al. (2014) which included 191 women with primary subfertility and 129 women with secondary subfertility demonstrated no statistically significant difference in sexual dysfunction between women with primary and secondary subfertility [26]. However, a Turkish study by Keskin et al. (2011), which included 122 and 51 women with primary and secondary subfertility respectively found a significantly lower individual and total FSFI scores in the secondary subfertile group [13].

We further sought to determine the association between the various sociodemographic variables and sexual dysfunction. Among the various socio-demographic variables, only education level and use of hormonal contraception were significantly associated with sexual dysfunction in the subfertility group and fertility control groups respectively. In the subfertility group, attainment of college level education appeared to be protective against sexual dysfunction. Higher education level has been shown to be protective of sexual dysfunction in other studies [19, 20]. This is possibly due to better health seeking behaviours associated with higher education. Use of hormonal contraception in the fertility control group was associated with a greater occurrence of sexual dysfunction. Previous similar studies yielded conflicting results. Fataneh et al. (2013) evaluated 608 married Iranian women aged 15–49 years (case group = 306 and control =302). The case group was those on contraception. The study showed a significant impairment in sexual function in the case group though only 26.8% were on hormonal contraception (pills). Moreover, those using barrier methods and vasectomy had a better sexual functioning [29]. In contrast, studies by Safarinejad (2006) and Li et al. (2004) failed to show any significant impairment of sexual function among women on various contraceptive methods [16, 20]. A systematic review by Pastor et al. (2013) that evaluated 36 studies (1978–2011; 13, 673 women) also found no significant impairment in sexual desire in women on combined oral contraceptive pill use [14]. The greater occurrence of sexual dysfunction in the present study would be attributed to the smaller study numbers and the fact that the study wasn’t powered to specifically evaluate for the association between contraceptive use and FSD.

Given that there was no significant difference in FSD in the subfertile and fertility control groups, the two groups were assessed together for factors with possible association with FSD. The variables found to be significantly associated to sexual dysfunction were maternal age and alcohol use. Maternal age above 35 years of age appeared to be protective (adjusted odds ratios 0.41, CI 0.18–0.91, *p* = 0.028). This is possibly due to the population bias and a possible better socioeconomic status of women above the age of 35 years. Moreover, they possibly have a better understanding of their own sexuality than the younger women. Alcohol use also appeared to be protective (adjusted odds ratio 0.40, CI 0.18–0.89, *p* = 0.024). Alcohol use possibly makes women more expressive of their sexual feelings hence less occurrence of sexual dysfunction.

Some of the limitations of the present study include that it was conducted in a tertiary private hospital whose clientele are generally of a higher education level and socioeconomic status and therefore, the results may not be generalizable to the general population. Secondly, given the sensitive nature of the subject matter, the study subjects might have been emotionally swayed in their responses to the questions. We also had little information regarding the women’s partners which may have affected their sexual function.

## Conclusions and recommendations

In conclusion, the present study demonstrated no association between the fertility status and the prevalence female sexual dysfunction. Subfertility type was not associated with sexual dysfunction. Education level and use of hormonal contraception were associated with sexual dysfunction in the subfertility and fertility control groups respectively while alcohol use and higher maternal age appeared to be protective against female sexual dysfunction. Given the limitations of the present study, we recommend a large multi-centre study in our setting to further evaluate the association between subfertility and sexual dysfunction.

## Data Availability

The datasets used and analyzed during the current study are available from the corresponding author on reasonable request.

## Abbreviations

BMI: Body Mass Index
CI: Confidence Interval
DSM-IV: Diagnostic and Statistical Manual of Mental Disorders, 4th edition.
FSD: Female Sexual Dysfunction
FSFI: Female Sexual Function Index
FSFI-Q: Female Sexual Index Questionnaire
SD: Standard Deviation
OR: Odds Ratio
